# Methylated (−)-epigallocatechin 3-O-gallate potentiates the effect of split vaccine accompanied with upregulation of Toll-like receptor 5

**DOI:** 10.1038/s41598-021-02346-4

**Published:** 2021-11-29

**Authors:** Motofumi Kumazoe, Kanako Takamatsu, Fuyumi Horie, Ren Yoshitomi, Hiroki Hamagami, Hiroshi Tanaka, Yoshinori Fujimura, Hirofumi Tachibana

**Affiliations:** 1 Division of Applied Biological Chemistry, Department of Bioscience and Biotechnology, Faculty of Agriculture, Kyushu University, 744 Motooka, Nishi-ku, Fukuoka, 819-0395 Japan; 2 Department of Chemical Science and Engineering, Tokyo Institute of Technology, 2-12-1 Ookayama, Meguro, Tokyo, 152-8552 Japan

**Keywords:** Biotechnology, Chemical biology, Immunology, Molecular medicine

## Abstract

Split-virus vaccine serves as a major countermeasure against influenza virus, but its effectiveness and protective action are not complete. We previously demonstrated the effect of Benifuuki, a green tea cultivar in Japan, on enhancing the split-virus vaccine–elicited immune response. However, little is known about the detail mechanisms. Here, we show that EGCG3”Me intake significantly potentiated the vaccine-elicited hemagglutination inhibition titer increase. Flow cytometry analysis revealed the increased Toll-like receptor 5 (TLR5) expression after EGCG3”Me treatment in lamina propria dendritic cells (LPDCs) and macrophages, which play crucial roles in the humoral immune system. TLR5 expression correlated with the level of interleukin-6 (IL-6)/C–C chemokine type receptor 5, which are important mediators of the humoral immunity. Taken together, In vivo and ex vivo studies showed that EGCG3”Me potentiated the split-virus vaccine–elicited immune response accompanied with the upregulation of TLR5 in intestine and splenocyte macrophages.

## Introduction

As many as 500,000 people die every year of influenza worldwide, and 3,000,000 to 5,000,000 cases of severe influenza A (*genus Alphainfluenzavirus*) and influenza B (*genus Betainfluenzavirus*) virus infections are reported^1^. Anti-viral agents are developed to target viral neuraminidase, a crucial enzyme involved in the vital process. However, the use of anti-viral agents is limited, and vaccination is the major countermeasure. Considering the risk/benefit balance, influenza prevention is preferred with split-virus vaccines (trivalent inactivated vaccine [TIV] and recent quadrivalent influenza vaccine [QIV]), which are better tolerated in patients^1^. However, the protective effect of split-virus vaccines is modest^2^. Previous meta-analysis based on 14 observational studies and 17 randomized trials have revealed the modest efficacy of a split-virus vaccine (calculated efficacy 59% [95% confidence interval, 51–67%] in adult between 18 and 65 years of age)^2^, and additional measures are strongly demanded.

In the complicated human immune system, the activation of innate immunity is indispensable for the development of acquired immunity. Ligand of pattern recognition receptors (PRRs) that recognize pathogen-associated molecular patterns (PAMPs) are limited in split-virus vaccine and may hinder its clinical efficacy.

Toll-like receptor 5 (TLR5), one of the PRRs against flagellin, was shown to play a crucial role in mediating the response of split-virus vaccines such as TIV influenza vaccine and IPOL™, a purified viral subunit vaccine against polio similar to TIV in regards to being an purified viral subunit vaccine^3^. TLR5 plays a crucial role in developing acquired immune response, including antibody production with split/purified viral subunit vaccines, but may be not necessary in the YF-17D, the live-attenuated yellow fever vaccine. These data indicate that TLR5 augments the innate immune signaling in the split/purified viral subunit vaccine-elicited immune response^3^. Consistent with these findings in humans, a significant correlation was reported between induced TLR5 expression and hemagglutination inhibition (HAI) titer at 28 days after vaccination^3,4^. However, no approach has been established to enhance TLR5 expression.

Antigen-presenting cells (APCs), including macrophages and dendritic cells, are important connectors between the innate and acquired immune systems. TLR5 expression on APCs is involved with antibody response^3^. After their activation by PAMPs, APCs secrete cytokines, including interleukin (IL)-6 and type I/II interferons (IFNs), all of which are associated with the activation of the acquired immune system.

Green tea is one of the most consumed beverages in the world. We have previously reported that Benifuuki, one of the green tea cultivars, significantly enhanced the split-virus vaccine-elicited immune response in vivo^5^. Considering the risk/benefit balance, this tea cultivar is commercially available, widely consumed for a long time, economic, and safe, making it a potential supplement to boost split-virus vaccine efficacy. However, the detailed mechanism of the effect of Benifuuki is not clarified.

Here, we showed that (**−**)-epigallocatechin 3-(3"-*O*-methyl) gallate (EGCG3”Me), a characteristic polyphenol in Benifuuki, upregulates TLR5 expression on macrophages and lamina propria dendritic cells (LPDCs). EGCG3”Me intake enhanced the split-virus vaccine-elicited immune response in vivo.

## Materials and methods

### Reagents

EGCG3”Me was synthesized by the group of Tokyo Institute of Technology. EGCG was obtained from Taiyo Kagaku (Mie, Japan). MF and AIN-93G (rodent standard diet) chow were purchased from KBT Oriental (Saga, Japan). The split-virus vaccine against influenza, receptor-destroying enzyme (RDE) (II) Seika, and HA antigen (A/California/7/2009, H1N1) were procured from Denka Seiken Co., Ltd (Tokyo, Japan). Chicken red blood cells were obtained from Wako (Osaka, Japan), and isoflurane (from Mylan Inc., (Tokyo, Japan). Collagenase D and DNase I were supplied by Roche (Tokyo, Japan), and Accudenz A.G. cell separation media were provided by Accurate Chemical & Scientific Corporation (Westbury, NY, United States).

### Animals and animal experiments

The research was performed in accordance with the regulations in Japan (no. 105) and notification (no. 6). All studies were approved by the Animal Care and Use Committee of Kyushu University, Fukuoka, Japan (A28-159). All procedures were carried out in accordance with guidelines. Because the effects of EGCG3”Me in mice were not predicted, group size was decided by previous study^5^. All mice were maintained in a temperature (20 °C) and humidity (60%) controlled room with a 12-h light–dark cycle (light from 8 AM to 8 PM). We confirmed that the study is reported in accordance with ARRIVE guidelines. In the baseline model, 11-week-old female BALB/c mice (n = 8/ 3 group total 24 in 3 group) were purchased from KBT Oriental (Saga, Japan) and acclimated for 1 week while being fed MF diet (Saga, Japan). Mice were randomly divided into 3 groups and administered vehicle (dH_2_O), EGCG (10 mg/kg p.o.) or EGCG3”Me (10 mg/kg p.o.) for 1 week in 2 cages per group and all mice were sacrificed under isoflurane vapor at the same day. The organs were harvested and used for analysis. In the split-virus vaccine model, titer is the primary outcome and the detail measurement procedure were described in the below, 11-week-old female BALB/c mice (n = 8/ 3 group total 24 in 3 group) were purchased from Kyudo (Saga, Japan), divided into 3 groups, and administered AIN-93G (rodent standard diet) from KBT Oriental (Saga, Japan) with or without EGCG3”Me (0.01%/diet; 20 mg/kg body weight) (Table 1) for 5 weeks in 2 cages per group. Mice were treated with split-virus vaccine (1 μg/mouse i.d.) at 2 and 4 weeks and then sacrificed at 5 weeks. All mice were sacrificed under isoflurane vapor on the same day. Blood (via retro-orbital sinus) and organs were harvested. Serum was collected after incubation (6 h, at 4 °C), centrifuged (1500 ×g for 15 min at 4 °C) and stored at − 80 °C. For HAI titer evaluation, 200 μL serum samples were obtained from the mice fed AIN-93G diet or EGCG3”Me-containing AIN-93G diet for 5 weeks and were incubated with 60 μL RDE (II) for 18 h at 37 °C. RDE was inactivated at 56 °C for 1 h and then diluted with phosphate buffer (4.25 g sodium chloride [NaCl], 0.7125 g disodium hydrogen phosphate [Na_2_HPO_4_]/12H_2_O, and 0.0675 g monopotassium phosphate [KH_2_PO_4_] in 500 mL dH_2_O). The samples (10 diluted) were treated with a 10 uL of chicken red blood cells at room temperature for 1 h and centrifuged at 900 ×g for 5 min. The supernatant was harvested and diluted to 10–1280 times, all diluted samples (25 μL) were added to a V-shaped 96-well plate and incubated with 4HA (A/California/7/2009, H1N1; 25 μL) for 30 min, followed by treatment with 0.5% chicken red blood cells for 1 h. Any aggregation observed was recorded.

**Table 1:** Normal diet and EGCG3”Me 0.01% containing diet.

Component	Normal diet (AIN-93G diet)	Normal diet + EGCG3”Me 0.01%
Vitamin mix	1	1
Mineral mix	3.5	3.5
Choline bitartrate	0.25	0.25
L-Cystine	0.3	0.3
Soybean oil	7	7
Tertiary butylhydroquinone	0.0014	0.0014
Granulated sugar	10	10
Milk casein	20	20
Α-Corn starch	13.2	13.2
Corn starch	39.7	39.7
Cellulose powder	5	4.99
EGCG3”Me	0	0.01
Total (%)	100	100

All animals were randomly assigned (chose alternately) to each group and not blinded. In vivo studies, the experimental unit is the mouse. Mice were provided drinking water and chow ad libitum.

### Antibodies and flow cytometry analysis

Fluorescein isothiocyanate (FITC)-labeled anti-mouse IgA antibody was purchased from Bio-Rad (Hercules, CA, USA), and Alexa Fluor 488-labeled anti-mouse CD11b was obtained from BD (Franklin Lakes, New Jersey). Allophycocyanin (APC)-labeled anti-mouse F4/80 was procured from Miltenyi Biotec (Rhine-Westphalia, Germany), and FITC-labeled anti-mouse CD11c and APC-labeled anti-mouse CD11b, from Miltenyi Biotec. Phycoerythrin (PE)-labeled anti-mouse TLR5 was supplied by Novus Biologicals (Centennial, CO, United States), and APC-labeled anti-mouse CD3 and FITC-labeled anti-mouse CD4 were provided by Miltenyi Biotec. PE-labeled anti-mouse Foxp3 were purchased from BD. All antibodies were diluted with 0.1% sodium azide and 2.5% bovine serum albumin (BSA) using a dilution buffer. Cytometry analysis was performed using Verse™ (BD). Flow cytometric histograms with smoothing function were depicted using a corresponding software. There is no excluded animal. In the LPDC flow cytometry study, data were subjected to an outlier test (ROUT test), and after outliers were removed, analyzed by Student’s t test.

### Reverse-transcriptase quantitative polymerase chain reaction (RT-qPCR)

Organs and cells were harvested and minced in TRI Reagent™ (500 μL) and mixed with 100 μL chloroform (CHCl_3_) for 3 min. The samples were centrifuged at 12,000 ×*g* for 15 min at 4 °C, and 200 μL of the harvested supernatant was incubated with 2-propanol (200 μL) for 10 min at room temperature. After the samples were centrifuged at 12,000 ×*g* for 5 min at 4 °C, the pellet was dried and diluted with nuclease-free water (NFW) (Ambion, TX, United States). RNA concentration was determined by Nano Drop 2000 (Waltham, MA, United States). RNA (100 ng/mL) was subjected to reverse transcription using the Prime Script™ RT Reagent Kit (Takara Bio, Shiga, Japan). A 1.4 μL cDNA template was added to the master mix (Sso Advanced™ Universal SYBR® Green Supermix [Bio-Rad], primers, and dH_2_O) and analyzed using CFX96™ Real-time PCR (Bio-Rad). The primers used were as follows: msb-Actin forward 5′-CATCCGTAAAGACCTCTATGCCAAC-3′ and reverse 5′-ATGGAGCCACCGATCCACA-3′; msTLR5 forward 5′-AAGGACAAGGTCTGGAGTTTGGA-3′ and reverse 5′-GCCTGTTTCGGGAACTGTAGTG-3′; msIL6 forward 5′-CCACTTCACAAGTCGGAGGCTTA-3′ and reverse 5′-GCAAGTGCATCATCGTTGTTCATAC-3′. msIFN-beta (MA114062)/msCCR5 (MA105386) primers were purchased from Takara Bio (Shiga, Japan).

### Cell cultures

In brief, 6-week-old female BALB/c mice (Kyudo) were sacrificed and their spleens were harvested. The splenocytes were minced in Roswell Park Memorial Institute (RPMI)-1640 medium (Nissui Pharma, Tokyo, Japan) using sterilized glass slides and filtered through a mesh. The cell suspension was centrifuged (1,300 ×*g*, 5 min) and washed with RPMI-1640 medium. The pellet was hemolyzed for 1 min on ice using a hemolysis buffer, and then harvested (1300 ×*g*, 5 min), washed with RPMI-1640 medium, and centrifuged (1,300 ×*g*, 5 min). Splenocytes were seeded at 2 × 10^6^ cells/mL in 10% fetal calf serum (FCS)-RPMI-1640 medium in a 12-well plate and treated with indicated concentrations of EGCG3”Me. After 24 h of treatment, cells were harvested using TRI Reagent.

### Dosage information

Mice were orally administrated with EGCG or EGCG3 “Me (10 mg/kg body weight) based on the formula for translation of dose by surface area from mouse to human as follows: human equivalent dose (HED) (mg/kg) = animal dose (mg/kg) multiplied by animal Km/human Km^7^. The HED for EGCG3 “Me is 0.81 mg/kg, which equates to a dose of 48.6 mg for a 60 kg person. This is equivalent to approximately 3 g of tea leaves^8^.

Mice were orally administrated with EGCG3“Me (20 mg/kg body weight) based on the formula for translation of dose by surface area from mouse to human as follows: HED (mg/kg)^7^. The HED for EGCG3“Me is 1.62 mg/kg, which equates to a dose of 97.2 mg for a 60 kg person. This is equivalent to approximately 6 g of tea leaves^8^.

### Statistical analysis

All values are expressed as means ± standard error of means (S.E.M) versus control groups. Statistical analysis was performed by the Student’s *t*-test using GraphPad Prism 8 software (San Diego, CA, United States).

## Results

### Analysis of the effect of EGCG3”Me on TLR5 expression in the spleen by flow cytometry

**Figure 1:**
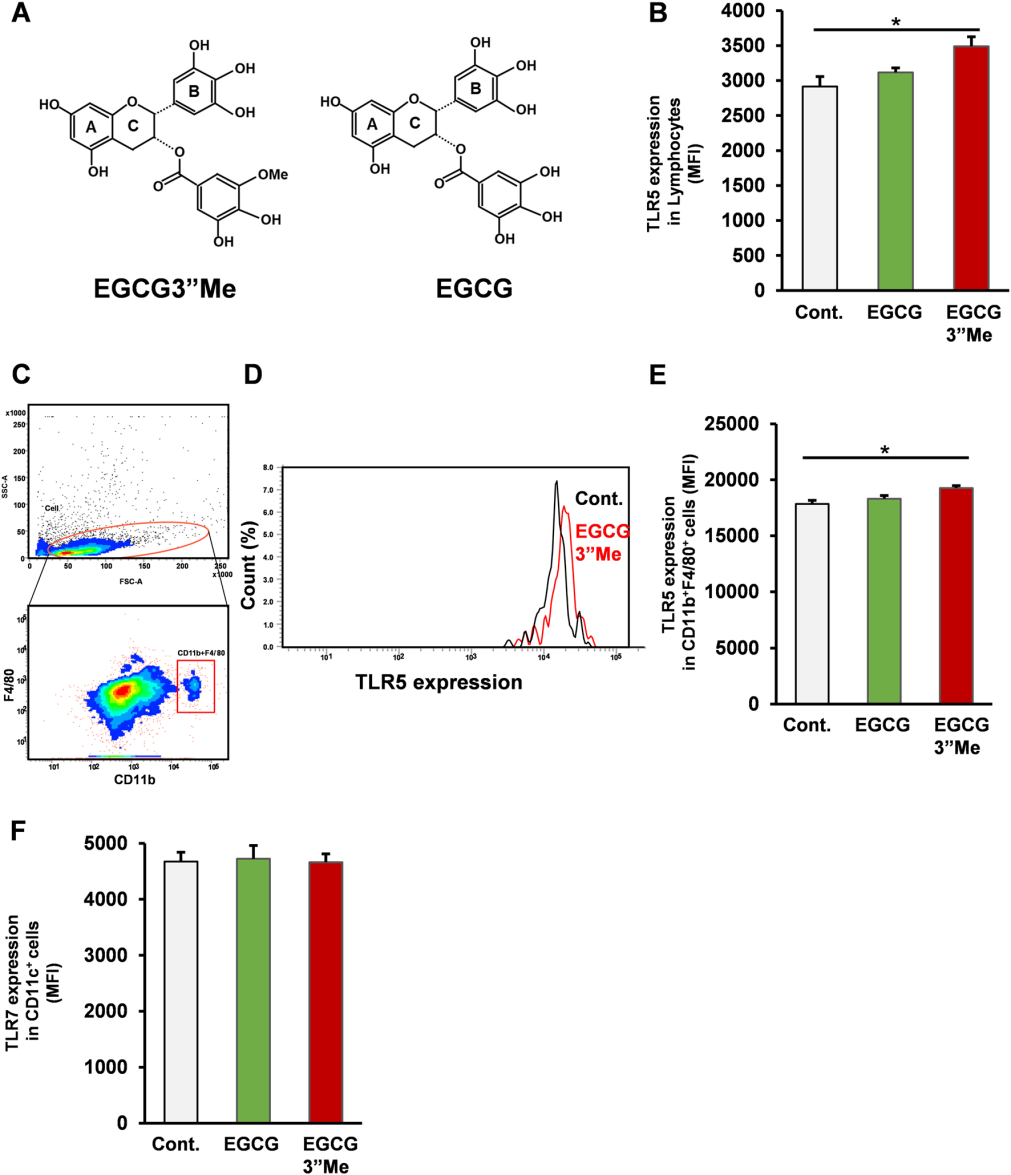
EGCG3”Me intake upregulated TLR5 expression in vivo. (**A**) The chemical structure of EGCG3”Me (**B**–**E**) 12-week-old female BALB/c mice were orally administrated with vehicle, EGCG (10 mg/kg intragastric administration), or EGCG3”Me (10 mg/kg intragastric administration) every day. After 1 week, mice were sacrifced and TLR5 expression was determined by flow cytometry (**B**) TLR5 expression in lymphocytes, (**C**–**E**) TLR5 expression in CD11b^+^ F4/80^+^ cells. (n = 8). (**F**) TLR7 expression was evaluated by flow cytometry (n = 8). Data are presented as means ± SEM ^*^P < 0.05.

An extract of the green tea cultivar Benifuuki potentiated the split-virus vaccine-elicited immune response in a mouse model. Here, we hypothesized that EGCG3”Me (Fig. 1A), the characteristic polyphenol in Benifuuki, upregulates TLR5 expression in vivo. To assess the effect of EGCG3”Me (equivalent to approximately 3 g green tea leaves based on HED^7,8^) intake on the mouse immune system, 12-week-old female BALB/c mice were orally administered vehicle (dH_2_O), EGCG (10 mg/kg intragastric administration) and EGCG3”Me (10 mg/kg intragastric administration) for 1 week. After treatment, mice were sacrificed under isoflurane vapor and their organs harvested. Flow cytometric analysis of CD11b^+^F4/80^+^ cells (macrophages) and whole splenocytes showed that EGCG, the major catechin in green tea, had no effect on TLR5 expression on these cells (Fig. 1B–E, two-tailed Student’s *t*-test vs control). In contrast, EGCG3”Me strongly enhanced TLR5 expression on both whole splenocytes and CD11b^+^F4/80^+^ cells (Fig. 1B–E, two-tailed Student’s *t*-test vs control, *P* < 0.05). The expression of TLR7, the sensor for single-strand RNA, on CD11c^+^ cells (dendritic cells) was affected neither by EGCG nor EGCG3”Me treatment (Fig. 1F, two-tailed Student’s *t*-test vs control). Taken together, the intragastric administration of EGCG3”Me enhanced TLR5 expression on both CD11b^+^F4/80^+^ cells (macrophages) and whole splenocytes.

### RTqPCR analysis of the effect of EGCG3”Me on TLR5 expression on splenocytes by RTqPCR

**Figure 2:**
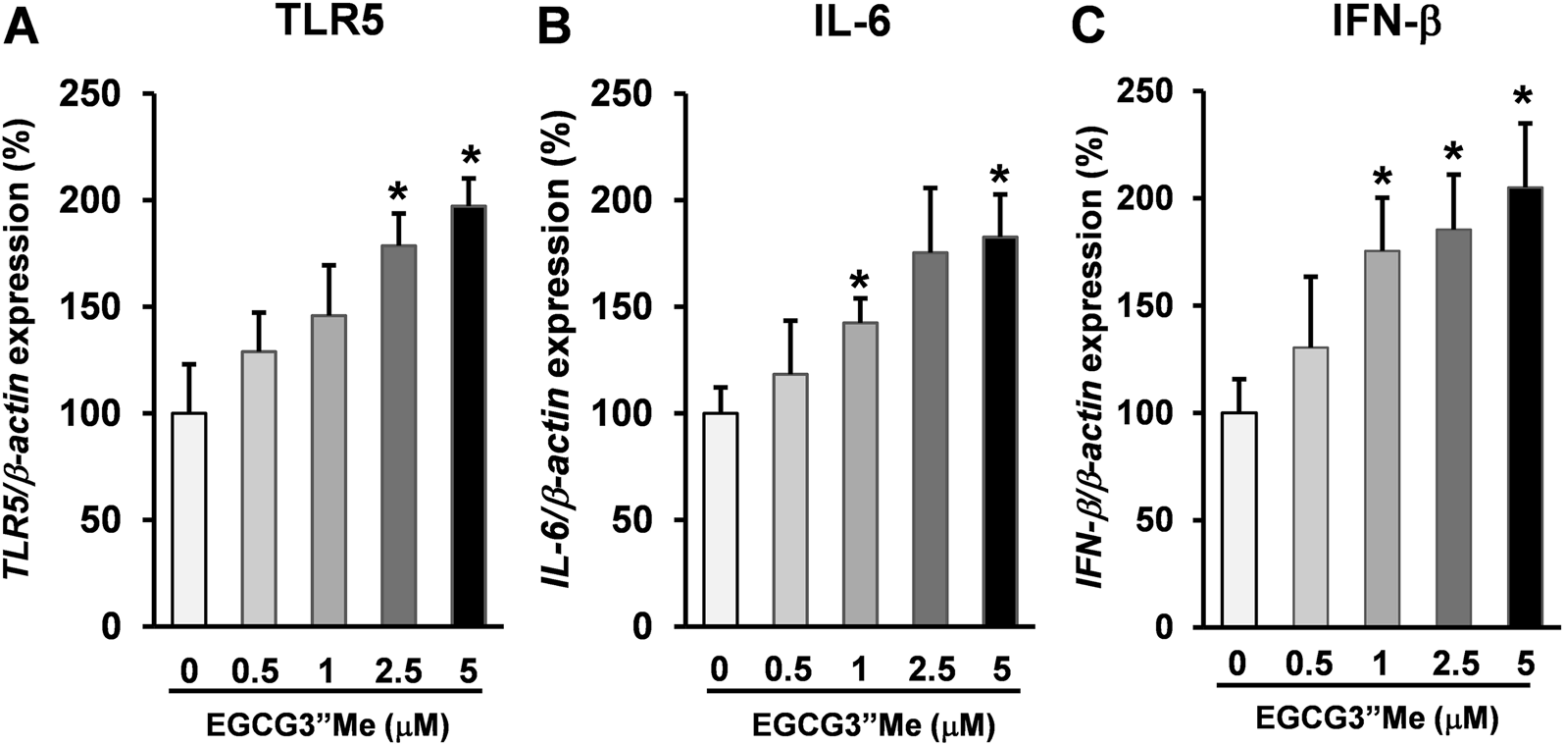
EGCG3”Me upregulated TLR5 expression on splenocytes. (**A**–**C**) Female BALB/c mice were sacrificed and the spleens were harvested. Splenocytes were seeded at 2 × 10^6^ cells/mL in 10% FCS-RPMI-1640 medium in 12-well plates and treated with different doses of EGCG3”Me for 24 h. After treatment, cells were harvested and the mRNA expression was evaluated by RT-qPCR. (**A**) TLR5 (n = 4), (**B**) IL-6 (n = 4), (**C**) IFN-beta (n = 4). Data are presented as means ± SEM ^*^P < 0.05 (vs control).

As EGCG3”Me is a highly absorbent green tea polyphenol^8^ with stronger bioactivity than EGCG^9^, we hypothesized that EGCG3”Me exerts direct pharmacological effects on splenocytes. To assess the direct effect of EGCG3”Me on TLR5, BALB/c mice were sacrificed and their spleen harvested. Splenocytes were seeded in 10% FCS-RPMI-1640 medium and treated with EGCG3”Me at different doses (0.5, 1, 2.5, and 5 μM) for 24 h. Cells were then harvested and the mRNA expression was evaluated by RT-qPCR. EGCG3”Me upregulated the expression of TLR5 on splenocytes in a dose-dependent manner (Fig. 2A, two-tailed Student’s *t*-test vs control, *P* < 0.05). A recent study showed that TLR5 plays a crucial role in IFN-β production^10^. Moreover, type I IFNs^11^ and IL-6^12^ participate in the acquired immune response. EGCG3”Me treatment also increased the expression of IL-6 in splenocytes in a dose-dependent manner, consistent with TLR5 expression (Fig. 2B, two-tailed Student’s *t*-test vs control, *P* < 0.05). In line with our previous findings, EGCG3”Me treatment upregulated the expression of IFN-β in splenocytes in a dose-dependent manner (Fig. 2C, two-tailed Student’s *t*-test vs control, *P* < 0.05). Taken together, EGCG3”Me may have a direct effect on splenocytes through the upregulation of TLR5 and its downstream signaling.

### EGCG3”Me enhanced the immune response elicited by split-virus vaccine in vivo

**Figure 3:**
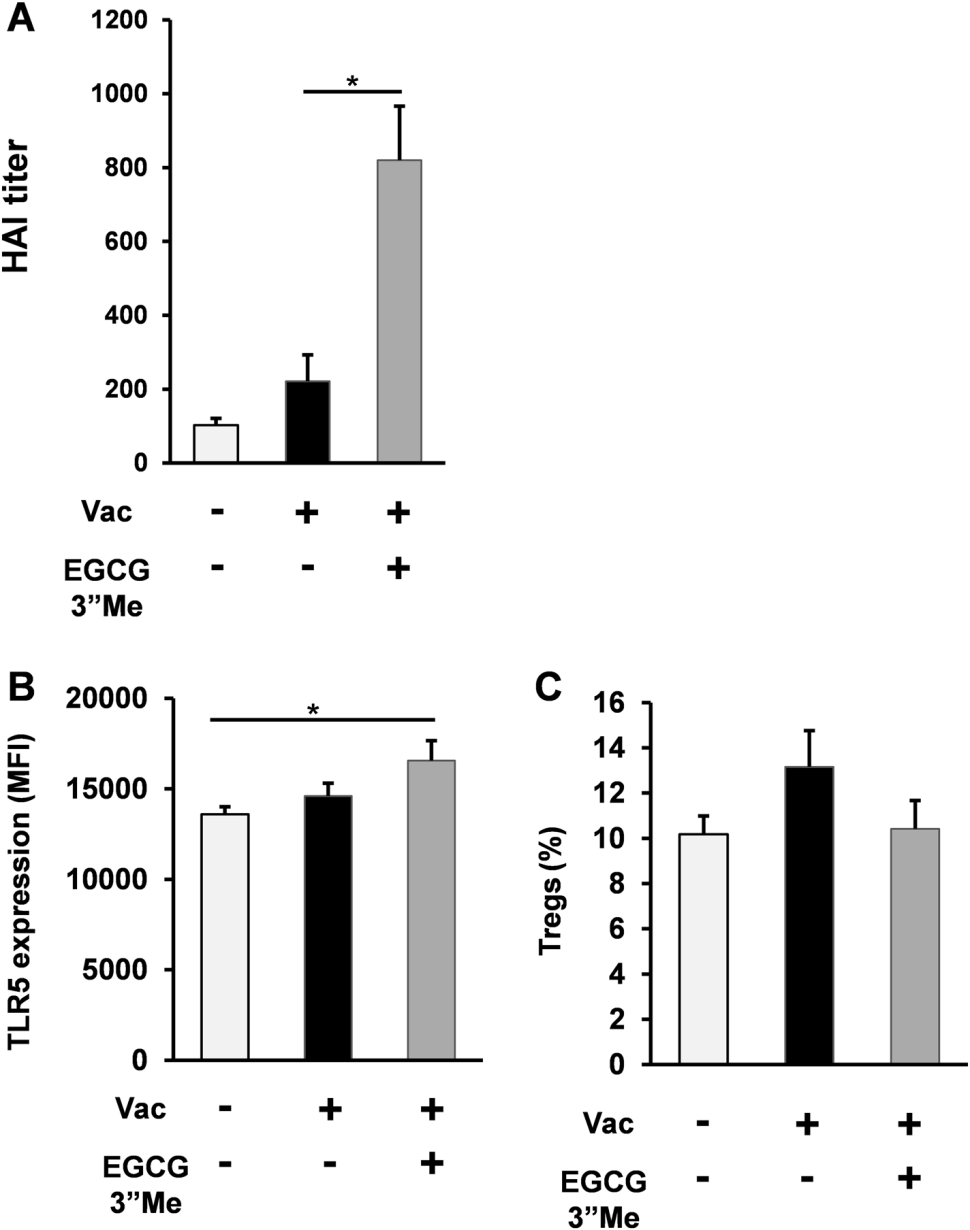
EGCG3”Me enhanced the immune response elicited by split-virus vaccine in vivo. (**A**) 12-week-old female BALB/c mice were fed AIN-93G diet or AIN-93G diet supplemented with 0.01% EGCG3”Me (EGCG3”Me 20 mg/kg body weight) for 5 weeks. Mice were treated with split-virus vaccine (1 μg HA/mouse i.d.) at 2 and 4 weeks and then sacrificed at 5 weeks. Blood samples were obtained and HAI titer was evaluated (n = 8). (**B**) TLR5 expression level on splenic macrophages was determined by flow cytometry analysis (n = 8). (**C**) Treg expression was evaluated by flow cytometry analysis (n = 8). Data are presented as means ± SEM ^*^P < 0.05.

The HAI assay is a well-established approach to evaluate the ability of inactivated influenza vaccine in clinical protection^13^. Clinical data demonstrated that the magnitude of the HAI titer 4 weeks after vaccination correlated with TLR5 expression upregulation in human subjects^4^. To assess the effect of EGCG3”Me on HAI titer, BALB/c mice were fed AIN-93G diet or AIN-93G diet supplemented with 0.01% EGCG3”Me (EGCG3”Me 20 mg/kg body weight) for 5 weeks. Mice were treated with split-virus vaccine (1 μg HA/mouse i.d.) at 2 and 4 weeks, and then sacrificed at 5 weeks under isoflurane vapor. Their blood and organs were harvested, and HAI titer was evaluated. EGCG3”Me-containing AIN-93G diet intake significantly potentiated the split-virus vaccine-elicited immune response (Fig. 3A, two-tailed Student’s *t-*test vs. control, *P* < 0.05). Consistent with the effect of EGCG3”Me on the spleen tissue, EGCG3”Me-fed mice showed increased expression of TLR5 on splenocyte macrophages, indicating that EGCG3”Me intake upregulated TLR5 expression on systemic APCs (Fig. 3B, two-tailed Student’s *t*-test vs negative control, *P* < 0.05). EGCG3”Me intake also tended to suppress Treg cells^+^, but no significant difference was observed (Fig. 3C, two-tailed Student’s *t*-test vs negative control).

### EGCG3”Me intake enhanced TLR5 expression in split-virus vaccine-treated mice

**Figure 4:**
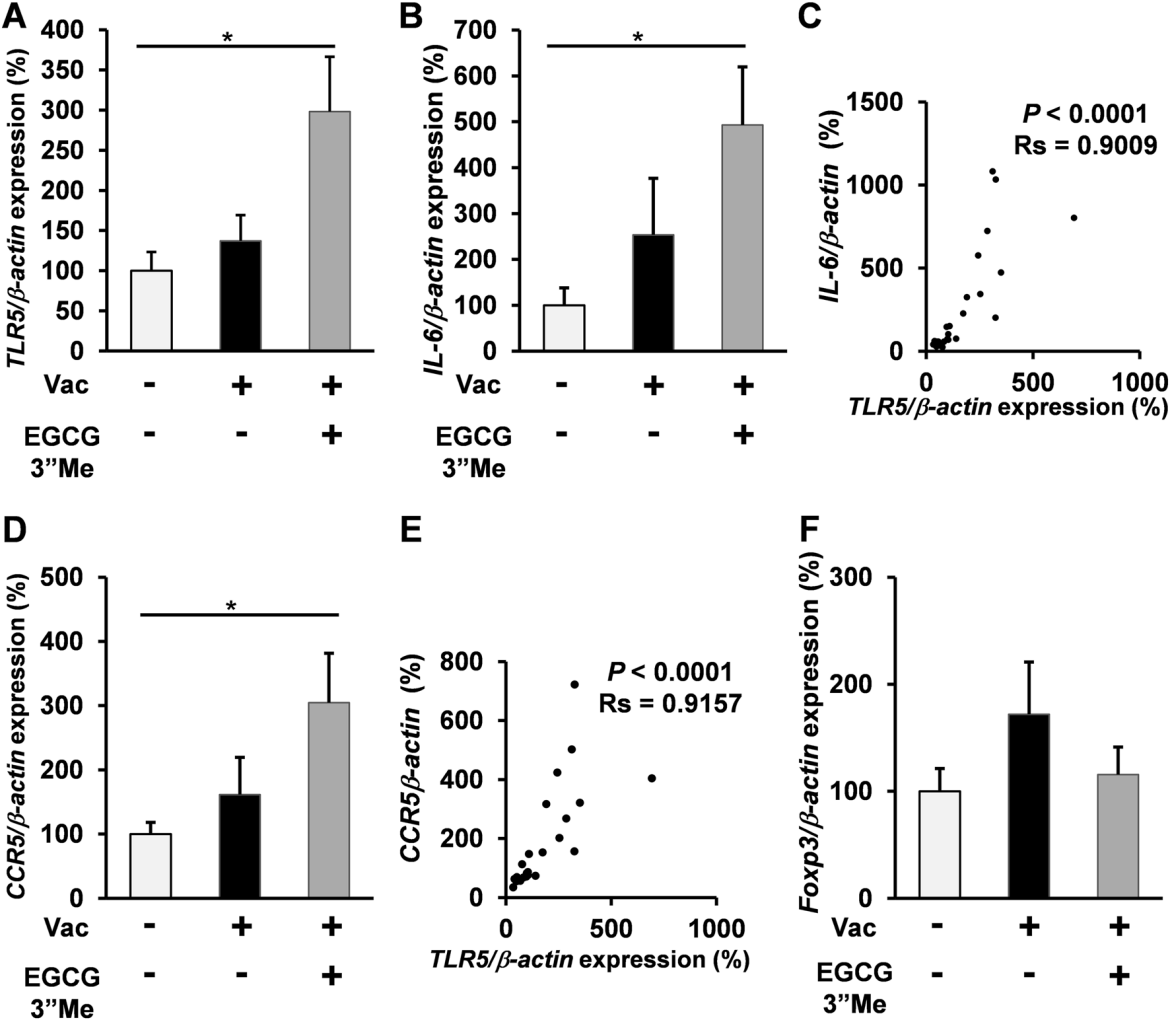
EGCG3”Me intake enhanced TLR5/IL6 expression in the intestine of split-virus vaccine-treated mice. (**A**–**F**) 12-week-old female BALB/c mice were fed AIN-93G diet or AIN-93G diet supplemented with 0.01% EGCG3”Me (EGCG3”Me 20 mg/kg body weight) for 5 weeks. Mice were treated with split-virus vaccine (1 μg HA/mouse i.d.) at 2 and 4 weeks. Mice were sacrificed at 5 weeks, and their intestines were harvested and evaluated for (**A**,**B**) TLR5 and IL6 expression by RT-qPCR (n = 8). (**C**) Correlation was analyzed by Spearman’s rank test (n = 24). (**D**) CCR5 expression was determined by RT-qPCR (n = 8). (**E**) Correlation was analyzed by Spearman’s rank test (n = 24). (**F**) Foxp3 expression was determined by RT-qPCR (n = 8). Data are presented as means ± SEM ^*^P < 0.05.

To confirm the effect of EGCG3”Me on the expression of TLR5 in the intestine, RT-qPCR was performed for samples obtained from EGCG3”Me-fed mice. EGCG3”Me significantly upregulated TLR5 expression as compared with the negative control treatment (Fig. 4A, two-tailed Student’s *t*-test vs negative control, *P* < 0.05), while the vaccine alone failed to upregulate TLR5 expression as compared with the negative control treatment. We also evaluated the expression of IL-6, acting downstream in TLR5 signaling^14^, which is associated with antibody response^15,16^. EGCG3”Me intake upregulated IL-6 expression as compared with the negative control treatment (Fig. 4B, two-tailed Student’s *t*-test vs negative control, *P* < 0.05), while the vaccine alone failed to induce any significant change in IL-6 expression as compared with negative control group. There is a significant correlation between expression of TLR5 and IL-6 (Spearman rank test, Rs = 0.9009, P < 0.0001, n = 24; Fig. 4C). Moreover, the C–C chemokine receptor type 5 (CCR5), closely related receptor for antibody responses^17^ is also upregulated by EGCG3”Me intake (Fig. 4D, two tailed Student’s *t*-test vs negative control, *P* < 0.05).; significant correlation was observed between TLR5 and CCR5 expression (Spearman’s rank test, Rs = 0.9157, *P* < 0.0001, n = 24; Fig. 4E). TLR5 is known to have an inhibitory effect on regulatory T cells (Tregs)^18^. Consistent with the previous findings, EGCG3”Me intake tended to suppress Foxp3 expression, but no significant effect was noted (Fig. 4F).

### EGCG3”Me intake induced immunoregulation TLR5 expression in LPDCs from split-virus vaccine-treated mice

**Figure 5:**
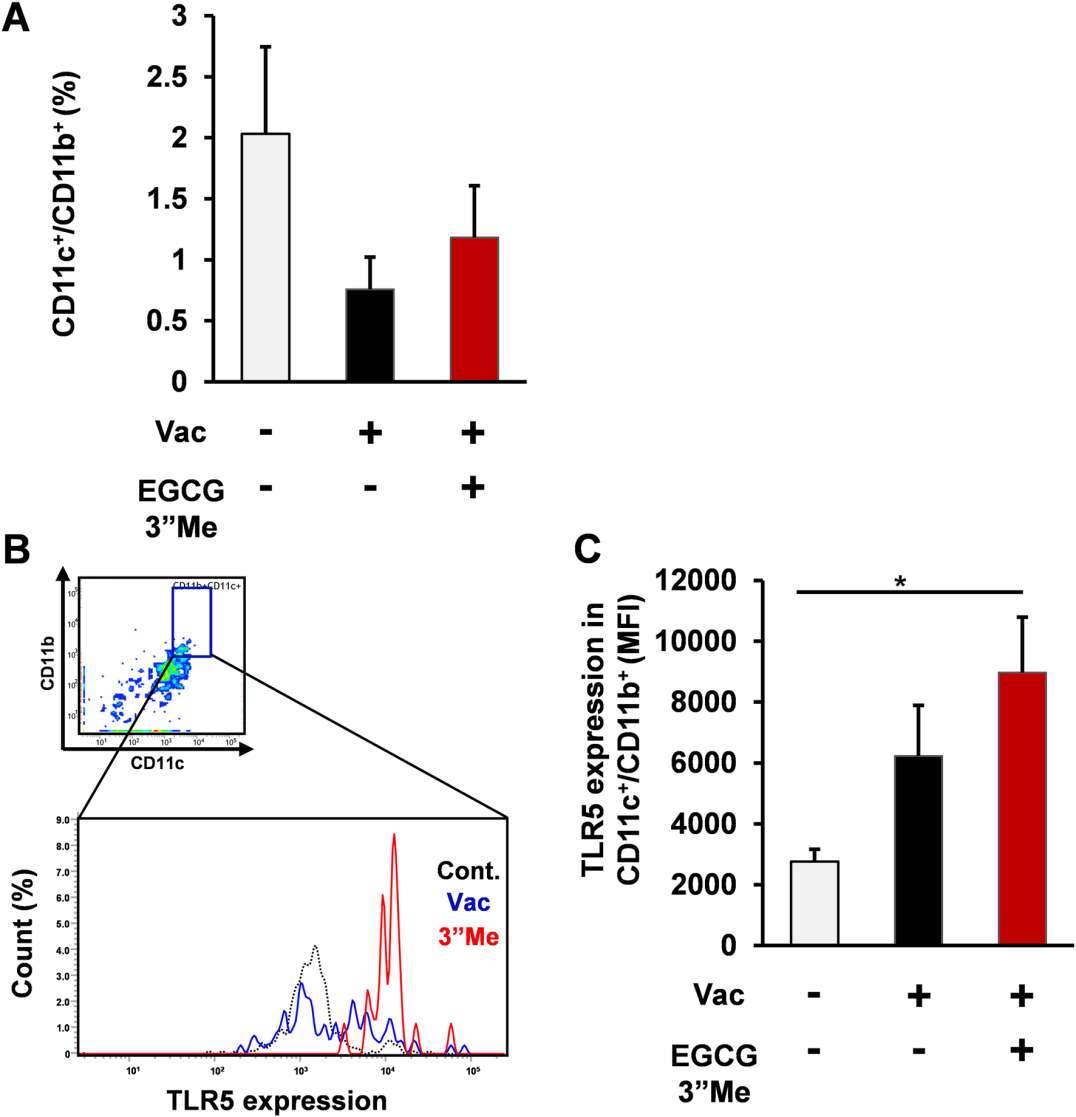
EGCG3”Me intake enhanced TLR5 expression on LPDCs and macrophages of split-virus vaccine-treated mice. (**A–E**) 12-week-old female BALB/c mice were fed AIN-93G diet or AIN-93G diet supplemented with 0.01% EGCG3”Me (EGCG3”Me 20 mg/kg body weight) for 5 weeks. Mice were treated with split-virus vaccine (1 μg HA/mouse i.d.) at 2 and 4 weeks and then sacrificed at 5 weeks. (**A**) CD11c^+^CD11b^+^ cells (**B, C**) and TLR5 expression on CD11c^+^CD11b^+^ cells were determined by flow cytometry (n = 6–8). Data are presented as means ± SEM *P < 0.05.

Flow cytometry analysis showed that EGCG3”Me intake did not change LPDCs (CD11b^+^CD11c^+^) (Fig. 5A) but increased TLR5 expression on LPDCs (CD11b^+^CD11c^+^), which are one of the major APCs involved in humoral immune response^19^, as compared with the negative control treatment (Fig. 5B, C, two-tailed Student’s *t*-test vs negative control, *P* < 0.05). Taken together, EGCG3”Me intake enhanced the split vaccine-elicited humoral immune response accompanied by immunoregulation of TLR5 expression. We have previously reported that Benifuuki, which is a methylated EGCG-containing green tea extract, enhanced the effect of split vaccine on the immune system, while Yabukita green tea extract (containing EGCG but not methylated EGCG) did not have a positive effect in enhancing the split vaccine-elicited immune response^5^. Consistent with our previous findings, we also showed that methylated EGCG induced immunoregulation of TLR5 in the intestine (Supplement Fig. 1 Mann Whitney U test, one tail).

## Discussion

Several clinical studies have demonstrated the efficacy of split-virus vaccines^20,21^, but the effect is not complete and further improvement is strongly demanded. Adequate innate immune signaling is essential to elicit acquired immune response^22^. However, there are not adequate PRR ligands in the split-virus and subunit vaccines. A recent study suggested that TLR5, a flagellin receptor, compensates for the innate immune stimuli through the microbiome^3^. Considering the importance of innate immune responses in the development of acquired immunity lacking in split-virus vaccines, TLR5 may be choke point in the split virus vaccine sensing. TLR5 knockout mice showed a drastically low sensitivity toward the split vaccine-elicited immune response but were responsive to live vaccines (that containing PAMPs)^3^. Consistent with these findings, TLR5 expression correlated with HAI titer in influenza split vaccine-treated humans^4^. However, no applicable approach has been established to upregulate TLR5 expression.

Here, we show for the first time that EGCG3”Me, the characteristic polyphenol in green tea cultivar Benifuuki, upregulated TLR5 expression on splenocytes, macrophages, intestines, and LPDCs, and enhanced the effect of the split vaccine in a mouse model. To the best of our knowledge, EGCG3”Me is the first orally delivered exogenous compound to upregulate TLR5 expression. EGCG3”Me enhanced the split-virus vaccine-induced HAI titer, a criterion used to evaluate protective effects of vaccines^13^.

In the prevention strategy, a risk/benefit balance is crucial. This tea cultivar is commercially available, widely consumed for a long time, economic, and safe, making it a potential supplement to boost split-virus vaccine efficacy. EGCG3”Me enhanced the split vaccine-elicited immune response accompanied with upregulating TLR5 expression. Moreover, green tea has no alcohol, sugar, or salt, and is consumed in many parts of the world. Considering these characteristics, Benifuuki could be a potent candidate to enhance the effect of split-virus vaccines.

The HED for EGCG3”Me (20 mg/kg body weight in mouse; split vaccine model) is 1.62 mg/kg body weight, equating to 97.2 mg/person (60 kg). This is amount of EGCG3”Me contained in 6 g green tea leaves^7,8^ and could be applicable in our daily life.

The microbiome is a hot topic in the healthcare industry. The microbiome has strong effects on obesity^23^, immunity^24^, vaccine efficacy^25^, and immune checkpoint inhibitors in cancer^26^. Many probiotics and prebiotics have been developed and are under development; however, the regulation of the sensing mechanisms is not well elucidated. Considering that TLR5 is a crucial PRR to “sense” the microbiome^27–29^, EGCG3”Me could serve as a potent enhancer for these probiotics and prebiotics because its long-term safety has been validated^30^. Our results provide a molecular basis for the development of novel probiotics and prebiotics.

We reported that EGCG3”Me exerts anti-inflammatory^31^, anti-obesity^9^, and anti-allergy effects^32^. The effect of EGCG3”Me on TLR5 seems paradoxical. O’Mahony D.S. et al. recently demonstrated that pro-inflammatory cytokines IFN-γ and granulocyte macrophage colony-stimulating factor (GM-CSF) upregulate TLR2 and TLR4 expression on monocytes but suppress TLR5 expression^33^. Considering the negative action of EGCG3”Me on TLR4-dependent signaling in macrophages^31^, these effects may be involved in the upregulation of TLR5.

A recent study indicated that protein kinase C (PKC) delta^ser664^ phosphorylation plays a crucial role in the expression of TLR5^34^. PKC inhibition downregulated TLR5 expression in HT29 cells. EGCG3”Me exhibits strong 67LR agonist activity^9,35^, and 67LR agonist phosphorylated PKC delta^ser664^ not only in multiple myeloma cells and chronic lymphoid leukemia^36,37^, but also in chronic myeloid leukemia^38^ and acute myeloid leukemia cells^39^. Considering that EGCG3”Me has favorable pharmacokinetic properties (five times higher area under the curve than EGCG^8^), these mechanisms also may contribute to the upregulation of TLR5 expression and warrant further studies.

Our *ex vivo* studies showed that EGCG3”Me upregulated TLR5 and IL-6 expression without affecting TNF-α expression (data not shown). EGCG3”Me exerts anti-inflammatory effects, but how it elicits IL-6 expression without PAMPs remains unclear. Recently, TLR5 has been identified as a receptor specific for damage-associated molecular patterns (DAMPs)^40^. However, the exact molecule is yet unknown. EGCG3”Me could possibly elicit IL-6 expression through the upregulation of TLR5 and increase the sensitivity to DAMPs.

We have previously reported that methylated EGCG retains its 67LR agonist activity^31^ and shows a superior pharmacokinetic character^8^. High absorbance and retention of 67LR agonist properties may contribute to the effects of methylated EGCG.

Previous studies have shown that green tea-inactivated influenza virus induced neutralizing antibodies against the virus^41^. Considering in our daily lives, these events are also involved in the influenza-prevention effect of green tea. Those studies have shown the direct interaction of EGCG and viruses, suggesting the increased effect of EGCG-treated viruses or EGCG-treated vaccine^41^. In this study, our setting was based on the potentiation of conventional vaccines. EGCG3”Me was administrated orally though the diet, while the split vaccine was administrated though injection.

Since we have previously reported the enhanced effect of Benifuuki (methylated EGCG containing green tea extract) on the split vaccine-induced immune response^5^, we focused on the effect of methylated EGCG on split vaccine-treated mouse in the present study. The antibody titer upregulating effect in EGCG3”Me and split vaccine in combination is drastic. The drug interaction between the EGCG3”Me and split vaccine is unclear because, to reveal the synergism, an isobologram analysis or combination index calculation is necessary, requiring many mice; this could not be performed due to ethical reasons. The limitation of our present study is that whether methylated EGCG had an additive or synergistic effect has not been determined. However, because a split vaccine consists of a viral antigen, methylated EGCG may enhance the antigen-elicited immune response.

Recent research has shown that the microbiome can regulate host immunes system functions^3^. Considering the importance of immune regulation in the microbiome^3^, the effect of EGCG3”Me on the immune system could be mediated by its effects on the microbiome. Previous reports have also suggested that TLR5 expression can regulate the microbiome^42^. Considering that EGCG3”Me upregulates TLR5 expression in the intestine, these processes are complicated and possibly interconnected. EGCG3”Me induces TLR5 expression in vitro, and this direct action may contribute to its effect. In conclusion, EGCG3”Me, the characteristic polyphenol in Benifuuki, increased TLR5 expression on macrophages and LPDCs in split vaccine-administrated mice, and enhanced the vaccine-elicited immune response *in vivo*. Considering that EGCG3”Me can be consumed through green tea cultivar Benifuuki, EGCG3”Me may be a good candidate to improve performance of split vaccine. Because TLR5 acts as a sensing molecule in the microbiome, EGCG3”Me could be a potent approach to increase the sensitivity of prebiotics and probiotics, thus demanding further clinical research.

## Supplementary Information

Supplementary Information.

## Abbreviations

APCs: Antigen-presenting cells
APC: Allophycocyanin
BSA: Bovine serum albumin
CCR5: C–C chemokine type receptor 5
DAMPs: Damage-associated molecular patterns
EGCG3”Me: (−)-Epigallocatechin 3-(3″-*O*-methyl) gallate
GM-CSF: Granulocyte macrophage colony-stimulating factor
FCS: Fetal calf serum
FITC: Fluorescein isothiocyanate
HAI: Hemagglutination inhibition
HED: Human equivalent dose
IL-6: Interleukin-6
LPDCs: Lamina propria dendritic cells
NFW: Nuclease-free water
PAMPs: Pathogen-associated molecular patterns
PE: Phycoerythrin
PKC: Protein kinase C
PRRs: Pattern recognition receptors
QIV: Quadrivalent influenza vaccine
RDE: Receptor-destroying enzyme
RPMI 1640: Roswell Park Memorial Institute-1640 medium
TIV: Trivalent inactivated vaccine
TNF-α: Tumor necrosis factor alpha
TLR5: Toll-like receptor 5
