# Supplementary material for: Methylated (−)-epigallocatechin 3-O-gallate potentiates the effect of split vaccine accompanied with upregulation of Toll-like receptor 5
Source: Sci Rep. 2021 Nov 29;11:23101. doi: 10.1038/s41598-021-02346-4 (PMC8630126; doi:10.1038/s41598-021-02346-4)
Supplement: Supplementary file 1 — Supplementary Information. [file 41598_2021_2346_MOESM1_ESM.pdf]

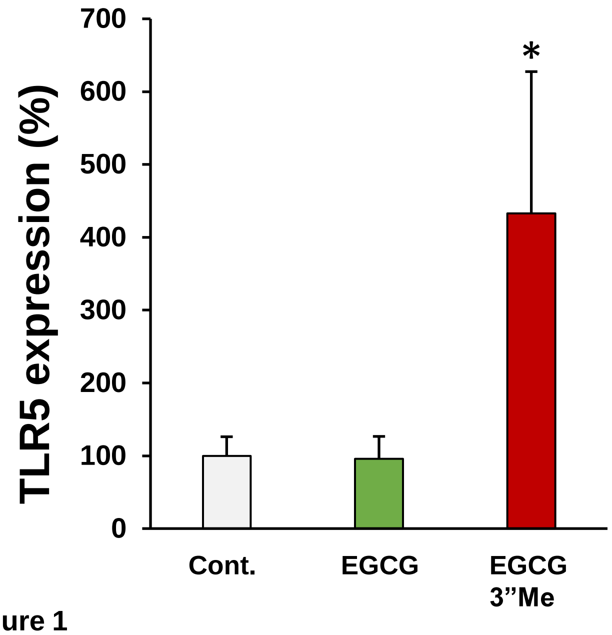

Supplementary Figure 1

Female balb/c mice (11 wk) were randomly divided into 3 group (I, Control group, II, EGCG group (10 mg/kg p.o.) and, III, EGCG3“Me group (10 mg/kg p.o.), each 6 mice) and treated with each compound for 1 week. Mice were sacrificed under isoflurane vapor. Data are presented as means ± SEM. *$P < 0.05$. Mann–Whitney U test, one tail.
